# The Hon. Arthur Stanley, C.B., M.V.O., M.P., Appointed Treasurer

**Published:** 1917-06-30

**Authors:** 


### ST. THOMAS'S HOSPITAL.

### The Hon. Arthur Stanley, C.B., M.V.O., M.P., Appointed Treasurer.

A Special General Court of the Governors of St. Thomas's Hospital was held in the Governors' Hall on the 27th instant at 3.15 p.m. The Archbishop of Canterbury attended and moved a vote of thanks "to J. G. Wainwright, Esquire, J.P., for the valuable services he has rendered to St. Thomas's Hospital as Treasurer during twenty-seven years," and subsequently the election of Mr. Wainwright as an extra member of Grand Committee for life. The Archbishop spoke warmly of Mr. Wainwright, whom he had known as a friend for many years, and to whose business gifts he testified by dwelling upon the financial strength the hospital had gained under Mr. Wainwright, not by mere good fortune, but as the result of good and continuous work. Twenty-seven years was a long period to fulfil the great and onerous duties of the Treasurership. A man of good age, when appointed to a post like this, would be liable in the course of these years to afford signs of senility. He might prove non-progressive in his policy, slow and stodgy in his grasp of pressing questions, and so end in becoming a fossil. Mr. Wainwright had avoided successfully all these dangers, and from the outset to the end the governors and officers and all associated with him found him to be invariably active and always progressive in his policy. Mr. Wainwright proved a good friend to St. Thomas's Hospital and all connected with it. He was always loyal to his fellow-workers and maintained the most cordial relations in co-operation with them. He exhibited a wonderful touch and knowledge when new developments were advocated by the younger men, and invariably voted for progress and the supply of the necessary funds to secure it. This was good for the hospital and the school, because the younger men were encouraged by the Treasurer's invariable buoyancy and good-will in a good cause. The more intimate the friendship between the Archbishop and the Treasurer became, the more the former realised how safe a leader and untiring a learner Mr. Wainwright invariably proved himself to be. St. Thomas's Hospital was deservedly known for the high tone and atmosphere of the hospital throughout. Since Mr. Wainwright became treasurer twenty-seven years ago he made it his business to know everything and everybody connected with St. Thomas's Hospital. Putting the best interests of the hospital, its rapid progress and development, first, he had proved himself to be in many respects an ideal treasurer and a good man.

Mr. A. I. Boyson, a member of the Grand Committee, seconded the resolution. He declared that Mr. Wainwright had no easy task when he assumed the office of Treasurer in 1890, for at that time affairs were in a somewhat chaotic condition, owing to the absence of administrative grip. Mr. Wainwright had given the best years of his life to St. Thomas's Hospital; his heart was in his work, which had never failed to give him satisfaction and pleasure from first to last. The Archbishop of Canterbury then read the following resolution which had been moved and seconded, and was carried unanimously.

Resolved.

That a record be entered on the minutes of the Court of Governors of this hospital in acknowledgment of the very valuable services rendered to the hospital by J. G. Wainwright, Esq., J.P., as Treasurer during the past twenty-seven years. Mr. Wainwright became a Governor in 1866 and served continuously as an Almoner from April 1874 until he was elected Treasurer in March 1890. From that time forward he devoted his life to the interests of the hospital, and it is due to his tact, diligence, and organising abilities that the hospital has reached its present high state of efficiency. Under his fostering care the work has increased, all the wards are now occupied, special departments to meet the ever-growing needs of medical and surgical knowledge and the requirements of out-patients have been organised, and the finances placed on a perfectly sound basis. To Mr. Wainwright's personal character the success of his period of office is largely due. He established the most cordial relations between the Governors and the staff, and has always succeeded in securing the enthusiasm and help of all the officers of the hospital. He has upheld them in their authority and supported them in all positions of difficulty, they have learned to trust him as their leader; and, inspired by his devotion to the hospital, they have given him a loyal and happy response.

Dr. Theodore Acland, who wore khaki, as representing the medical staff of the hospital expressed on behalf of his colleagues their deep feeling of gratitude to the late Treasurer for his invaluable service and cordial co-operation for twenty-seven years, and their sincere regret that the time should have arrived when Mr. Wainwright felt it necessary that he should resign his office, though every one of the staff rejoiced to know that they would still retain him as a friend and colleague.

Mr. Wainwright made an admirable speech, terse, full of feeling, and to the point. He insisted that he had only occupied the position of stroke in the boat, the victory of which was due to the efficiency and quality of the crew, his colleagues in the work. He expressed his gratitude for the invariable courtesy and co-operation of all grades connected with the hospital from first to last, and of the joy it had been to him to have had the responsibility of administering its affairs for so long a period of his life. Turning to the Governors, and much moved, he concluded with the words "From my heart I thank you."

The New Treasurer.

The Right Hon. the Lord Mayor proposed that the Hon. Arthur Stanley, C.B., M.V.O., M.P., be elected Treasurer of St. Thomas's Hospital. He dwelt upon the important position occupied by the Derby family, and the high respect in which every member of it was held throughout the country.

Members of the family had rendered great national service on several occasions, and Mr. Stanley's eldest brother was, as everybody knew, Secretary of State for War. The Lord Mayor expressed the pleasure it afforded him to testify to the high business gifts possessed by Mr. Arthur Stanley, with whom he had been associated since his residence at the Mansion House in connection with important matters relating to the war, and the care of and provision for the wounded. He was a most excellent chairman. The fact that he had since the war successfully held the important post of chairman of the Joint War Committee of the British Red Cross Society made him, in the opinion of the Governors and the City, a man who was exceptionally qualified for the post vacated by Mr. Wainwright. Mr. Stanley had been largely responsible for the provision and quality of the accommodation and treatment provided through the Red Cross for the relief and comfort of our wounded sailors and soldiers. St. Thomas's Hospital had already treated, in special wards erected for the purpose, upwards of 6,000 wounded soldiers and 860 officers. Mr. Stanley would therefore have an excellent opportunity to utilise his exceptional experience by assuming the administrative direction of one of the oldest, noblest, and most famous of endowed hospitals. He had every confidence and great pleasure in supporting the election of Mr. Stanley.

Mr. Deputy Thomas seconded the resolution and endorsed everything that the Lord Mayor had so eloquently said in support of it. The Governors of St. Thomas's Hospital were fortunate in having the opportunity of appointing Mr. Arthur Stanley as their treasurer, a feeling which was unanimously shared throughout the City.

The resolution was then put and carried with acclamation.

Mr. Stanley dwelt upon the feeling of responsibility which animated him in accepting the important post to which he had that day been appointed. Mr. Wainwright had spoken of himself as the stroke, and he might remind him that the success of the stroke of a winning boat qualified him for the post of coach when he relinquished that position. He should therefore welcome Mr. Wainwright as the coach to whom he could constantly refer and so secure a new friend who, he knew from experience, would prove most helpful and welcome. His association through the Red Cross with the care of the sick and wounded, and his closer connection with British nursing through the College of Nursing, now in course of organisation, had filled him with the ambition to devote his time to the promotion of everything which could tend to improve and further the treatment of the sick, and the status, independence, and advancement of British nurses. The beneficence of the public, who had so generously contributed the funds requisite to make the Red Cross organisation efficient and adequate to the claims upon it, made him hopeful and confident that our hospitals and nursing service would not be allowed to languish for want of funds. He had had experience in getting money, and he had had the experience of not getting it; so he felt himself equipped to face the responsibility of his new office with a stout heart. St. Thomas's Hospital could claim that it contained all that is best and all that is highest in British nursing. In connection with it was the Memorial to Florence Nightingale, a shrine at which the whole nursing world worshipped. He looked forward to the provision of an even finer memorial in the successful development of British Nurses and Nursing in the widest and best meaning of the words.

